# SummArIzeR: simplifying cross-database enrichment result clustering and annotation via large language models

**DOI:** 10.1093/bioinformatics/btag102

**Published:** 2026-02-28

**Authors:** Marie Brinkmann, Michael Bonelli, Anela Tosevska

**Affiliations:** Division of Rheumatology, Department of Internal Medicine III, Medical University of Vienna, 1090 Vienna, Austria; Division of Rheumatology, Department of Internal Medicine III, Medical University of Vienna, 1090 Vienna, Austria; Division of Rheumatology, Department of Internal Medicine III, Medical University of Vienna, 1090 Vienna, Austria

## Abstract

**Motivation:**

Enrichment analysis across multiple databases often results in a high level of redundancy due to overlapping terms, complicating the interpretation of biological data. To address this, we developed SummArIzeR, an R package to cluster and annotate enrichment results across multiple databases, enabling fast, intuitive interpretation and comparison across multiple conditions. SummArIzeR clusters enrichment results based on shared genes, calculates a pooled *P*-value for each cluster and facilitates the cluster annotation using large-language models. It further allows an easily interpretable visualization of the results.

**Results:**

Compared to existing tools, SummArIzeR provides unbiased and fast cluster annotation using large language models. We demonstrate that SummArIzeR achieves clustering comparable to manual curation while offering superior grouping based on shared underlying genes.

**Availability and implementation:**

The SummArIzeR package is available as an open-source R package, with a comprehensive user manual provided in its GitHub repository: https://github.com/bonellilab/SummArIzeR.

## 1 Introduction

Multi-OMICs techniques are powerful tools that allow the exploration of multiple biological layers, from the genome to the transcriptome and epigenome (Dai and Shen 2022, Hayes *et al.* 2024). These techniques generate large amounts of data that, after correct analysis and interpretation, can give meaningful insights into biological mechanisms and their perturbations. Despite the multitude of existing methods for analysis of such data, the interpretation remains a significant challenge, especially when more layers of information are integrated. Gene-set databases, such as **Gene Ontology (GO)** and **MSigDB**, have helped remarkably by enabling gene-set enrichment analysis and linking gene-sets to biological contexts (The Gene Ontology Consortium 2019, Bhuva *et al*. 2025).

In order to understand biological mechanisms, it’s often valuable to combine enrichment results from multiple databases. Yet, this can introduce redundancy, with many overlapping or similar terms, which can create inflated and complex visualization.

**Figure 1:**
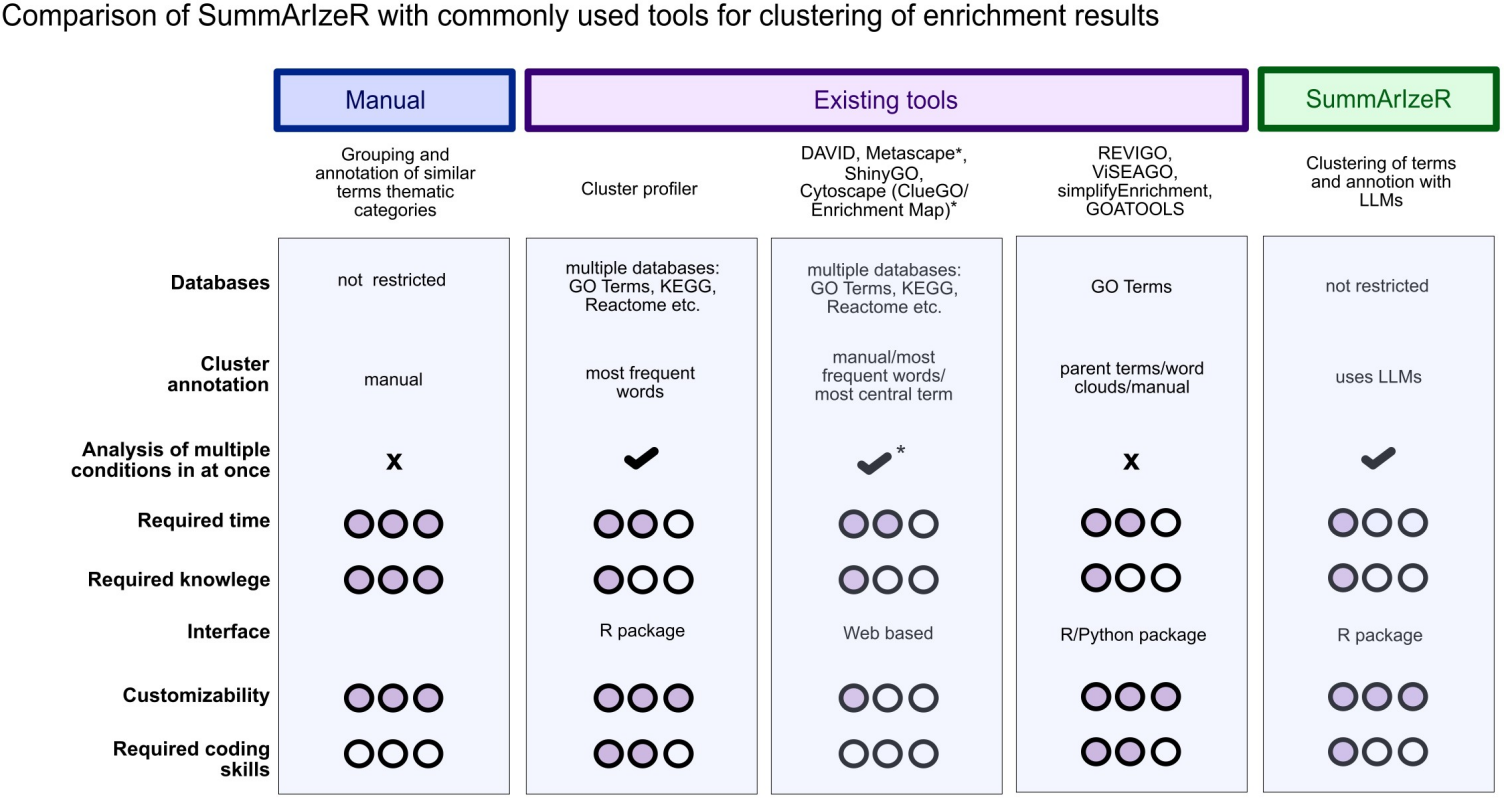
Comparison of SummArIzeR with existing tools for clustering enrichment results.

A variety of methods and tools have been developed to address this issue (Fig. 1). Traditionally, manually clustering and annotating these terms requires expertise and intuition and relies on term semantic similarity rather than shared underlying genes, which can often lead to misleading interpretations. While flexible as it is not limited to specific databases, this method is time-consuming, requires substantial biological expertise, and does not consider the underlying gene sets associated with each term.

Several clustering-based solutions, such as **REVIGO**, **VISEAGO**, **simplifyEnrichment**, and **GOATOOLS**, improve this by grouping similar GO terms based on term semantic similarity. However, these tools are generally restricted to Gene Ontology terms and can only process one condition at a time (Supek *et al.* 2011, Klopfenstein *et al.* 2018, Brionne *et al*. 2019, Gu and Hübschmann 2023).

To extend to multiple databases and conditions, web-based tools like **DAVID**, **Metascape**, **ShinyGO**, and **Cytoscape** offer user-friendly interfaces. Although these platforms are easily accessible, the analysis is time-consuming, and their output figures are often limited in customization (Bindea *et al.* 2009, Zhou *et al.* 2019, Ge *et al*. 2020, Sherman *et al.* 2022). For users comfortable with coding, **clusterProfiler** offers an R-based solution with more flexibility, but it requires a certain level of programming experience (Xu *et al.* 2024).

While all these tools address clustering to some degree, annotation of the resulting clusters remains limited. Most rely on parent term relationships or word-cloud-based algorithms, both of which can lose important biological information (Fig. 1) (Bindea *et al.* 2009, Supek *et al.* 2011, Klopfenstein *et al.* 2018, Brionne *et al*. 2019, Zhou *et al.* 2019, Ge *et al*. 2020, Sherman *et al.* 2022, Gu and Hübschmann 2023, Xu *et al.* 2024).

The rise of large language models (LLMs) like ChatGPT offers new possibilities in bioinformatics. LLMs have already begun supporting NGS and multi-OMICs data analysis, such as assisting in cell-type annotation for single-cell sequencing datasets (Hou and Ji 2024, Sarumi and Heider 2024, Enhancing functional gene set analysis with large language models 2025). They now present an exciting opportunity: unbiased, fast, and biologically informed annotation of multiple enrichment terms.

Here, we introduce SummArIzeR, an R package designed to streamline enrichment analysis across multiple conditions and databases. SummArIzeR clusters enriched terms based on shared genes and uses LLMs such as ChatGPT to generate meaningful cluster annotations, allowing us to investigate biological insights of OMICs data. We believe that SummArIzeR overcomes the aforementioned limitations. It enables simultaneous analysis of multiple conditions, such as different cell types, disease states, or treatment groups and supports the separate evaluation of up- and downregulated genes (Fig. 1). With a simple R interface requiring only minimal coding knowledge, users can quickly perform analyses.

## 2 Methods

SummArIzeR was developed using R 4.2.2, utilizing the **Enrichr** (Xie *et al.* 2021)**, dplyr**, **tidyr**, **stringr**, and **igraph** packages. Output plots are based on **Complex Heatmap** (Gu *et al*. 2016) and ggplot2. Additional plots were generated using **ggVennDiagram**.

### 2.1 Methods within SummArIzeR

#### 2.1.1 Enrichment analysis

Input gene lists are processed to extract gene identifiers and, optionally, log2 fold-change values. Functional enrichment is performed using either the **Enrichr** Application Programming Interface (API), or an offline available enrichment algorithm based on the hypergeometric test used in Enrichr. This offline functionality allows the user to select and upload custom gene sets. Results are filtered based on an adjusted *P*-value threshold of <.05 and a minimum number of genes per term. When specified, genes are separated into upregulated and downregulated subsets based on a user-defined log2 fold-change threshold.

To support the analysis of multiple experimental conditions simultaneously, the enrichment pipeline is applied across combinations of user-defined experimental groupings and selected **Enrichr** databases. Enrichment results from all conditions and databases are aggregated, with a configurable filter applied to retain the top-ranking terms per condition based on statistical significance.

#### 2.1.2 Clustering of terms

Identified terms are clustered based on methodologies previously implemented in the **simplifyEnrichment** package (Gu and Hübschmann 2023). Briefly, a binary gene-term incidence matrix is constructed to calculate pairwise Jaccard distances. A weighted undirected graph is built, and low-weight edges are pruned based on a user-defined threshold. Clusters are defined by the Community Walktrap algorithm (**igraph** package), allowing assignment of biologically coherent clusters. In brief, the walktrap community detection is based on random walks through a network in order to measure distance between vertices, where a random walk gets trapped in denser neighborhoods of the network that correspond to communities (Pons and Latapy 2005).

#### 2.1.3 Cluster annotation and *P*-value pooling

Generated clusters are annotated, by creating a prompt for any LLM. *P*-values for terms within clusters are pooled using a choice of four different methods: Fisher’s method (Fisher 1992), Stouffer’s method (Stouffer *et al*. 1949), a weighted Z-test (Zaykin 2011) or the Cauchy combination test (Liu and Xie 2020). By default, pooled *P*-values are capped at a minimum threshold for visualisation purposes.

Clusters get annotated by mapping manual summaries to cluster IDs. For each cluster and condition, *P*-values are pooled using the selected method. If regulation status (up/down) is available, it is retained. Redundant columns are removed, and unique terms and gene counts are calculated per cluster.

#### 2.1.4 Visualization: heatmaps and bubble plots

Enrichment results can be visualized using heatmaps and bubble plots. Heatmaps show −log10(pooled *P*-values), with optional bar plot annotations for term counts per cluster. Furthermore, conditions and clusters can be further grouped using hierarchical clustering. Bubble plots display condition vs. cluster annotation, with bubble size representing significance and color reflecting the number of genes enriched in the specific cluster.

### 2.2 Example data

To demonstrate the usage of SummArIzeR and compare it to published results, we used published data from Kugler *et al.* (2023). Data were processed as described in Kugler *et al*. and used as an input for SummArIzeR. GO terms were further used as an input for simplifyEnrichment v.1.99.0 (Gu and Hübschmann 2023) and rrvgo v. 1.20.0. (Supek *et al.* 2011) using the following parameters: similarity method “Sim_XGraSM_2013” and clustering method “binary_cut” for simplifyEnrichment; similarity method “Rel,” similarity threshold of 0.7 for rrvgo.

We additionally demonstrated the usability of SummArIzeR by applying it to publicly available datasets. Specifically, Della Chiara *et al.* performed clustering of KEGG pathways using the EnrichmentMap plugin in Cytoscape (Della Chiara *et al.* 2021), while Feng *et al.* clustered KEGG pathways and Gene Ontology Biological Process terms using the ClueGO Cytoscape plugin (Feng *et al.* 2022). For each study, we reproduced the corresponding enrichment analyses and extracted the reported sets of enriched terms for downstream comparison. SummArIzeR clustering thresholds (*ts*) were selected to reproduce the same number of clusters reported in the original publications.

We further compared the cluster annotation approach used by Feng *et al.*, which relied on parent terms, with SummArIzeR-based annotation and a word cloud–based annotation derived from the **simplifyEnrichment** package (Gu and Hübschmann 2023).

### 2.3 Comparison of clustering methods

Term membership within clusters was evaluated using the Jaccard index (Bonchi *et al*. 2013), calculated between the gene set of each individual term and the combined gene sets of the remaining terms within the same cluster. For each term, Jaccard indices were computed to quantify gene set overlap. These values were visualized as heatmaps, and the mean Jaccard index across all terms was compared between different clustering methods to assess overall cluster coherence (for clusters containing more than one term).

## 3 Results

### 3.1 SummArIzeR Workflow

**Figure 2:**
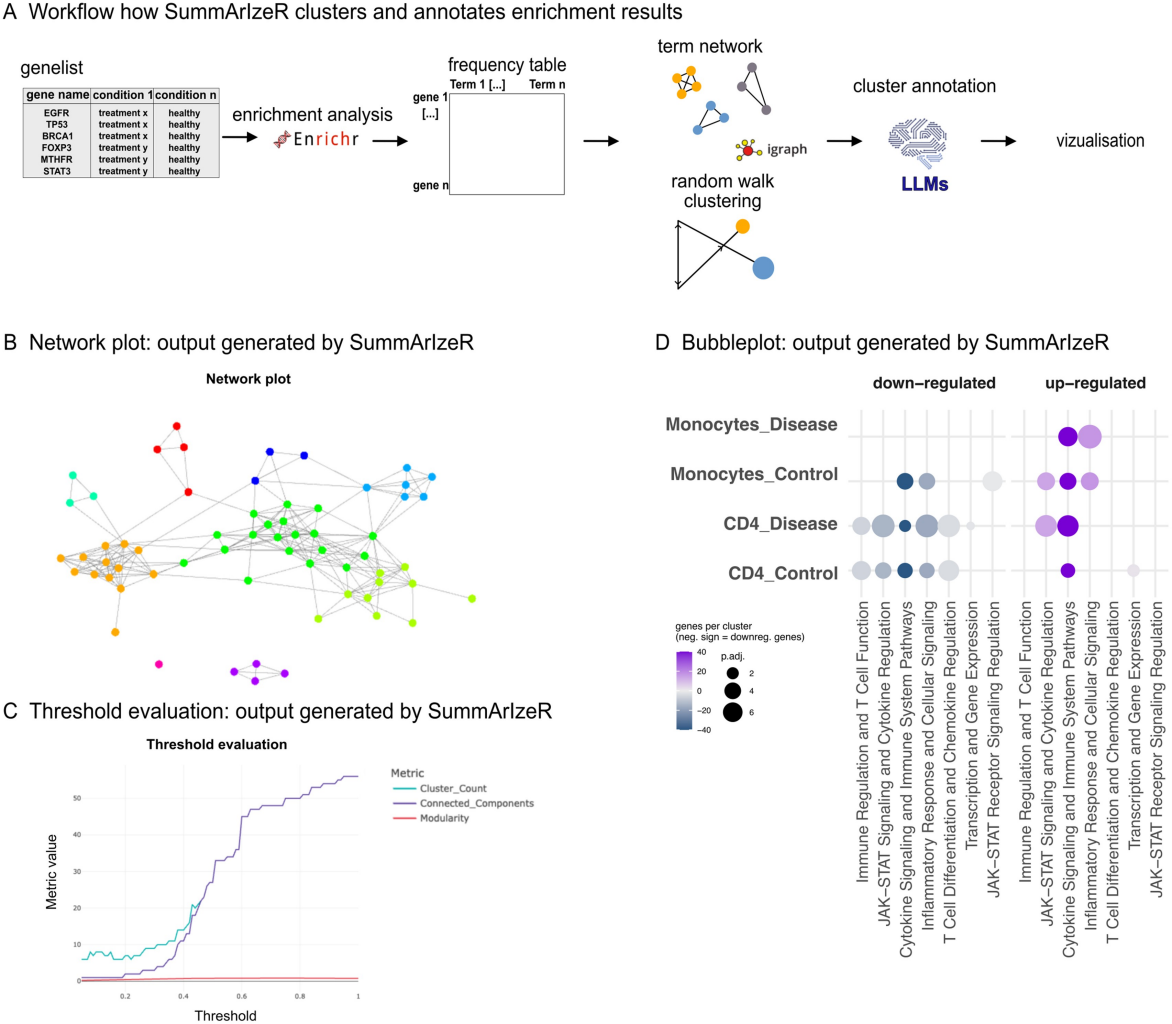
(A) Core workflow of SummArIzeR. (B) Example of a network plot (SummArIzeR output) with colors indicating cluster membership. (C) Threshold evaluation plot (SummArIzeR output) showing cluster count, number of connected components, and modularity change with varying thresholds. (D) Bubble plot (SummArIzeR output) generated with synthetic data, using the databases: GO_Biological_Process_2023, Reactome_2022, and BioPlanet_2019.

In a first step, users can upload a table including gene names and condition names (Fig. 2A). For a separate analysis of up- and downregulated genes, a column indicating log2-fold change has to be present. The user can select any database available from the extensive Enrichr library (Xie *et al.* 2021) or use custom gene sets with an internal enrichment function. The clustering can be influenced by setting a threshold parameter. An interactive igraph network visualizes the term clustering (Fig. 2B), and a threshold evaluation plot can be used to check modularity, connected cluster and cluster count for different thresholds (Fig. 2C). A unique feature of SummArIzeR is its use of LLMs to annotate clusters based on the underlying terms, preserving individual term information in an efficient and unbiased way (Figs 1 and 2A). The output is a table, listing clusters, their annotations, associated terms, and underlying genes. This avoids a “black box” approach, empowering users to investigate clustering decisions and customize their own visualizations. Graphical outputs include heatmaps and bubble plots (ggplot2 object), enabling straightforward comparison across multiple conditions (Fig. 1D).

### 3.2 Dataset: cytokine stimulation of fibroblast-like synoviocytes (FLSs) induces unique and shared transcriptional programmes.

**Figure 3:**
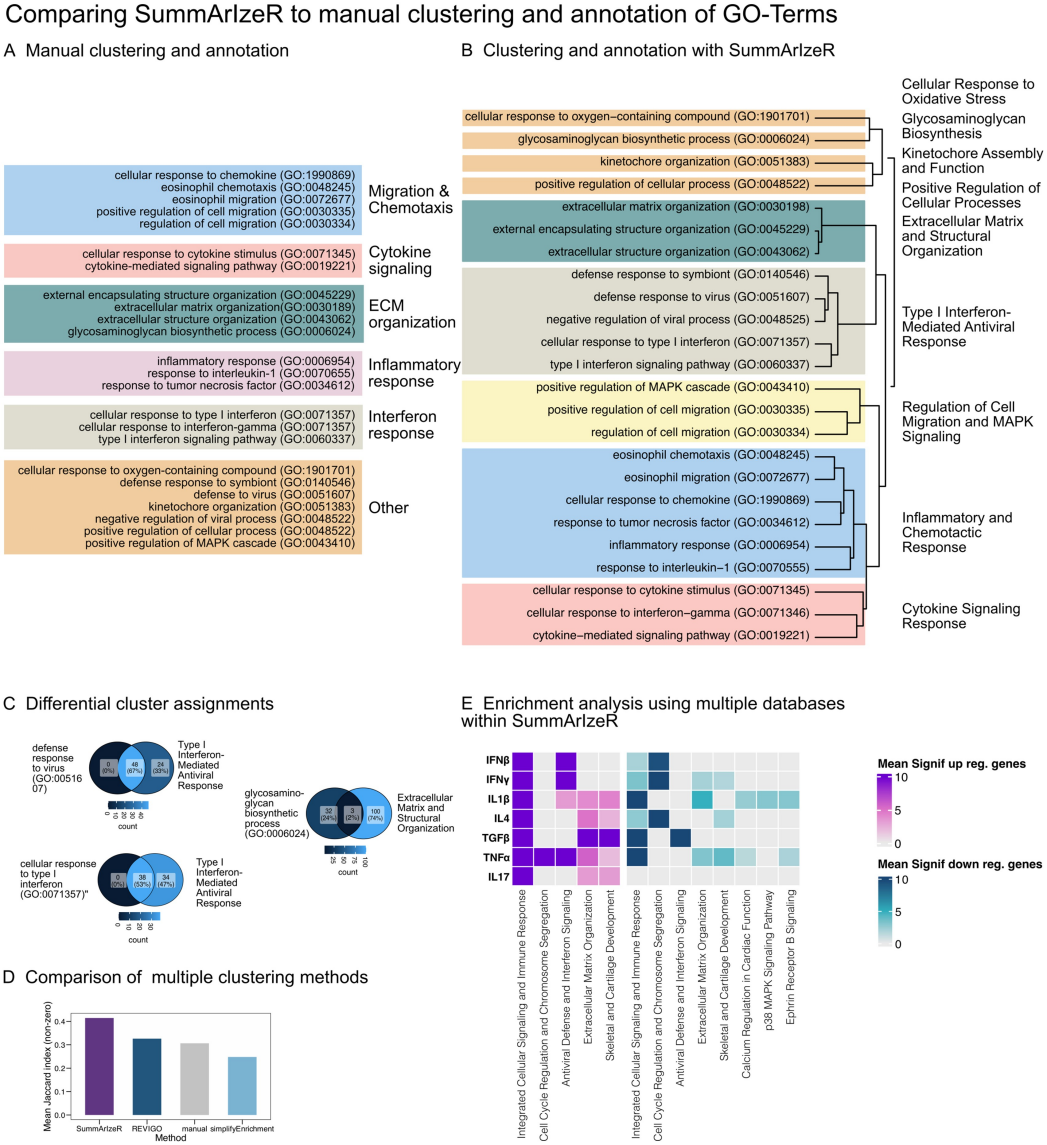
(A) Manual clustering of GO terms, following the approach used in Kugler *et al*. (2023, Fig. 2): The top five enriched terms per condition (based on the GO_Biological_Process_2021 database) were manually grouped. (B) Automated clustering using SummArIzeR with the same database (GO_Biological_Process_2021) and top five hits per condition. (C) Examples of differing cluster compositions, illustrated with Venn diagrams showing gene overlap between terms and clusters generated with SummArIzeR, visualized as color gradients. (D) Benchmarking of SummArIzeR clustering against manual clustering and existing methods (REVIGO and simplifyEnrichment). Cluster coherence was quantified using the mean Jaccard index, calculated for each term as the overlap between its gene set and the gene sets of the remaining terms within the same cluster (clusters containing more than one term). (E) Re-analysis of the dataset using SummArIzeR with different databases (GO_Biological_Process_2025 and BioPlanet_2019), based on the top five hits per condition (Heatmap visualization).

To demonstrate how SummArIzeR improves the visualization and interpretation of enrichment results, we applied it to a previously published dataset by our group that required comparison across multiple conditions and was affected by high term redundancy, requiring manual annotation (Kugler *et al.* 2023). The dataset contains bulk-RNA sequencing results of fibroblast-like synoviocytes, stimulated with different cytokines. We performed the enrichment analysis using the “GO-Biological-function_2021” database, selecting the top five terms per condition, comparable to the analysis performed in the publication (Kugler *et al.* 2023, Fig. 2). In the original analysis, terms were manually clustered into 6 groups based on term semantic similarity. With SummArIzeR, we performed the enrichment analysis under the same conditions. We observed similar cluster distribution; however, some differences could be observed in the term assignments (Fig. 3A and B).

The term “defense response to virus (GO:0051607)” was not assigned to a group in the original analysis (Fig. 3A), but it was assigned to the “Type I Interferon Mediated Antiviral Response” cluster by SummArIzeR. The enrichment analysis showed that 48 genes in the dataset matched the term. All of these genes overlapped with enriched genes found in the cluster “Type I Interferon Mediated Antiviral Response” after excluding the term “defense response to virus (GO:0051607)” from this cluster (Fig. 3C).

The term “glycosaminoglycan biosynthetic process (GO:0006024)” was manually clustered in the group “ECM organization” (Fig. 3A). In contrast, SummArIzeR clustered the term separately. Investigating overlapping genes revealed that only 2% of the genes overlapped with the cluster “Extracellular Matrix and Structural Organization,” after excluding the term “glycosaminoglycan biosynthetic process (GO:0006024)” from the cluster (Fig. 3C).

The term “cellular response to type I interferon (GO:0071357)” was manually annotated in a group called “Interferon response,” together with the term “cellular response to interferon-gamma (GO:0070655)” (Fig. 3A). The enrichment analysis showed that 38 genes in the dataset matched the term. All of these genes overlapped with enriched genes found in the cluster “Type I Interferon Mediated Antiviral Response” after excluding the term “cellular response to type I interferon (GO:0071357)” (Fig. 3C).

Overall, SummArIzeR exhibited higher within-cluster gene overlap compared to manual clustering. We next assessed SummArIzeR’s performance relative to established methods for GO term summarization, such as REVIGO and simplifyEnrichment (Fig. 1A and B, available as supplementary data at *Bioinformatics* online). To quantify cluster coherence, we computed the Jaccard index for each term, measuring gene set similarity between individual terms and their respective clusters. REVIGO and simplifyEnrichment produced clustering results comparable to SummArIzeR for some clusters, such as “Extracellular Matrix and Structural Organization.” In contrast, for selective terms as “defense response to symbiont” and “defense response to virus,” as well as for the “Inflammatory and Chemotactic Response*”* cluster, SummArIzeR achieved superior clustering performance compared with the other methods (Fig. 1C, available as supplementary data at *Bioinformatics* online). These differences were reflected in the mean Jaccard index across all clustered terms (clusters containing more than one term), which was highest for SummArIzeR (Fig. 3D).

As a next step, using SummArIzeR, we could extend the enrichment analyses to include other databases and visualize them (Fig. 3E). We used the “GO_Biological_Process_2025” and “BioPlanet_2019” databases, selecting the top 5 hits for each condition. We performed the cluster annotation with ChatGPT-4 and could further show that other LLMs like DeepSeek, Claude, Perplexity AI and Gemini AI led to similar cluster annotation (Table 1, available as supplementary data at *Bioinformatics* online). Including additional databases uncovered additional insights, such as the involvement of cell cycle-related genes, cartilage development pathways, calcium regulation mechanisms, and ephrin receptor B signaling - processes relevant to fibroblast activation in synovial tissue. Importantly, separating upregulated and downregulated genes revealed more nuanced patterns; e.g. TGF-β stimulation led to both upregulation and downregulation of genes involved in cellular signaling and immune responses, highlighting the complex regulatory dynamics.

To confirm the broader usability of the tool, we evaluated SummArIzeR across different datasets. We observed term clustering results comparable to the Cytoscape (EnrichmentMap)-based clustering reported by Della Chiara *et al.*, who performed enrichment analysis on enhancer-associated genes. While overall cluster structures were similar, SummArIzeR further resolved one cancer associated cluster into two distinct clusters: “Developmental and cancer-associated signaling pathways” and “Oncogenic signaling and cancer metabolism.” This refined separation resulted in higher Jaccard indices for the included terms and added an additional layer of biologically meaningful interpretation (Fig. 2B and C, available as supplementary data at *Bioinformatics* online).

In addition to term clustering, SummArIzeR provides an unbiased approach for cluster annotation. While SummArIzeR clustering was identical to the Cytoscape(ClueGO)-based clustering reported by Feng *et al.*, cluster annotation in that study relied on parent terms, which often misses important information when the parent and child terms are semantically unrelated (Table 2, available as supplementary data at *Bioinformatics* online). We therefore further compared this strategy to SummArIzeR and word cloud–based annotation.

SummArIzeR enabled informative cluster annotation while preserving information from all included terms. For example, the terms “HIF-1 signaling pathway,” “Renal cell carcinoma,” and “Central carbon metabolism in cancer” clustered together. Parent term–based annotation labeled this cluster as “HIF-1 signaling pathway,” whereas SummArIzeR annotation described it as “Cancer Metabolism and Hypoxia Signaling,” and word cloud–based annotation resulted in “cancer/carbon/carcinoma/cell/central.” Notably, only SummArIzeR annotation retained the biological information conveyed by each individual term.

The presented results could clearly demonstrate that SummArIzeR is a powerful tool for enrichment analysis, enabling systematic, unbiased clustering and annotation of enriched terms. It supports efficient visualization and direct comparison of multiple conditions, facilitating accurate and comprehensive data interpretation.

## 4 Discussion

To date, no tool has provided an easy and systematic approach for clustering and annotating enrichment results across multiple databases. With SummArIzeR, we introduce a user-friendly R-package that enables grouping of enrichment terms into clusters, annotation of these clusters, and direct comparison of *P*-values across multiple conditions.

SummArIzeR offers several advantages over manual annotation and existing tools. It provides an unbiased, reliable, and user-friendly approach, generating outputs that support fully customizable visualisation. We compared clustering and annotation results obtained with SummArIzeR to those generated through manual grouping of GO terms. SummArIzeR produced comparable clustering and annotations, while offering additional advantages by grouping terms based on shared underlying genes, even when term names did not directly overlap. Compared to the existing R packages REVIGO and simplifyEnrichment, SummArIzeR showed overall highest gene similarity between clusters. Furthermore, SummArIzeR revealed a more comprehensive enrichment result compared to the original analysis. It performed enrichment analysis separately for each condition and independently for upregulated and downregulated genes, ensuring that important results, especially from conditions with lower enrichment signals, are not overlooked.

Using additional publicly available datasets, we demonstrated that SummArIzeR enhances enrichment analysis and cluster annotation, thereby facilitating more straightforward and biologically meaningful interpretation.

SummArIzeR allows for easy and reliable enrichment based on a widely used tool such as Enrichr, while also adding offline functionality and customizability by allowing for user-provided enrichment gene sets. This functionality allows for broader application of the tool to data from different model and non-model organisms, as well as other omics analysis such as proteomics or metabolomics.

While LLMs facilitate cluster annotation, clustering more than 200 terms may occasionally result in incomplete output. This limitation is expected to improve as more advanced LLM versions become available. However, SummArIzeR’s transparent use of LLM outputs allows users to review and potentially adapt the generated annotation results.

Overall, we believe that SummArIzeR simplifies enrichment analysis across multiple databases and conditions, making it a valuable and practical addition to the R toolkit for sequencing data interpretation.

## Supplementary Material

btag102_Supplementary_Data

## Data Availability

The SummArIzeR package (v0.1.0) is available as an open-source R package, with a comprehensive user manual provided in its GitHub repository (2). All versions of the software are archived on Zenodo (28). The full source code and example datasets used to reproduce the analyses are openly accessible through the GitHub repository: https://github.com/bonellilab/SummArIzeR.
