# Supplementary material for: SummArIzeR: simplifying cross-database enrichment result clustering and annotation via large language models
Source: Bioinformatics. 2026 Feb 28;42(3):btag102. doi: 10.1093/bioinformatics/btag102 (PMC13005729; doi:10.1093/bioinformatics/btag102)
Supplement: btag102_Supplementary_Data [file btag102_supplementary_data.docx]

**SummArIzeR:** Simplifying cross-database enrichment result clustering and annotation via large language models

*Marie Brinkmann^1^, Michael Bonelli^1^, Anela Tosevska^1^*

^1^Division of Rheumatology, Department of Internal Medicine III, Medical University of Vienna, Austria

**Supplementary data**

**Supplementary table 1:** Cluster annotation (Figure 2D), comparing different large language models.

| **Cluster** | **ChatGPT-4 Turbo** | **DeepSeek** | **Claude** | **Perplexity AI** | **Gemini** |
| --- | --- | --- | --- | --- | --- |
| 1 | Skeletal and Cartilage Development | Skeletal and Muscular System Development | Skeletal and Cartilage Development | Skeletal and Musculoskeletal System Development | Skeletal and Muscle Development |
| 2 | Integrated Cellular Signaling and Immune Response | Inflammatory and Immune Signaling Pathways | Cytokine Signaling and Inflammatory Response | Immune Response and Intracellular Signaling Regulation | Immune and Inflammatory Responses and Signaling |
| 3 | Cell Cycle Regulation and Chromosome Segregation | Mitotic Cell Cycle and Chromosome Segregation | Mitotic Cell Cycle Regulation | Mitotic Cell Cycle and Spindle Assembly Regulation | Mitotic Cell Cycle and Spindle Organization |
| 4 | Antiviral Defense and Interferon Signaling | Antiviral Defense and Interferon Signaling | Antiviral Immune Response | Antiviral Defense and Interferon-Mediated Immune Signaling | Antiviral Defense and Interferon Signaling |
| 5 | Calcium Regulation in Cardiac Function | Calcium Ion Regulation and Cardiac Muscle Contraction | Calcium Ion Regulation | Regulation of Calcium Signaling and Cardiac Muscle Contraction | Regulation of Calcium Ion Release in Muscle Contraction |
| 6 | Extracellular Matrix Organization | Extracellular Matrix Organization | Extracellular Matrix Organization | Extracellular Matrix Organization and Collagen Pathways | Extracellular Matrix Organization |
| 7 | Ephrin Receptor B Signaling | Ephrin Receptor Signaling | Ephrin Signaling | Ephrin Receptor Signaling Pathway | Ephrin Receptor B Signaling |
| 8 | p38 MAPK Signaling Pathway | p38 MAPK Signaling Pathway | p38 MAPK Signaling | p38 MAPK Signaling Pathway | p38 MAPK Signaling |


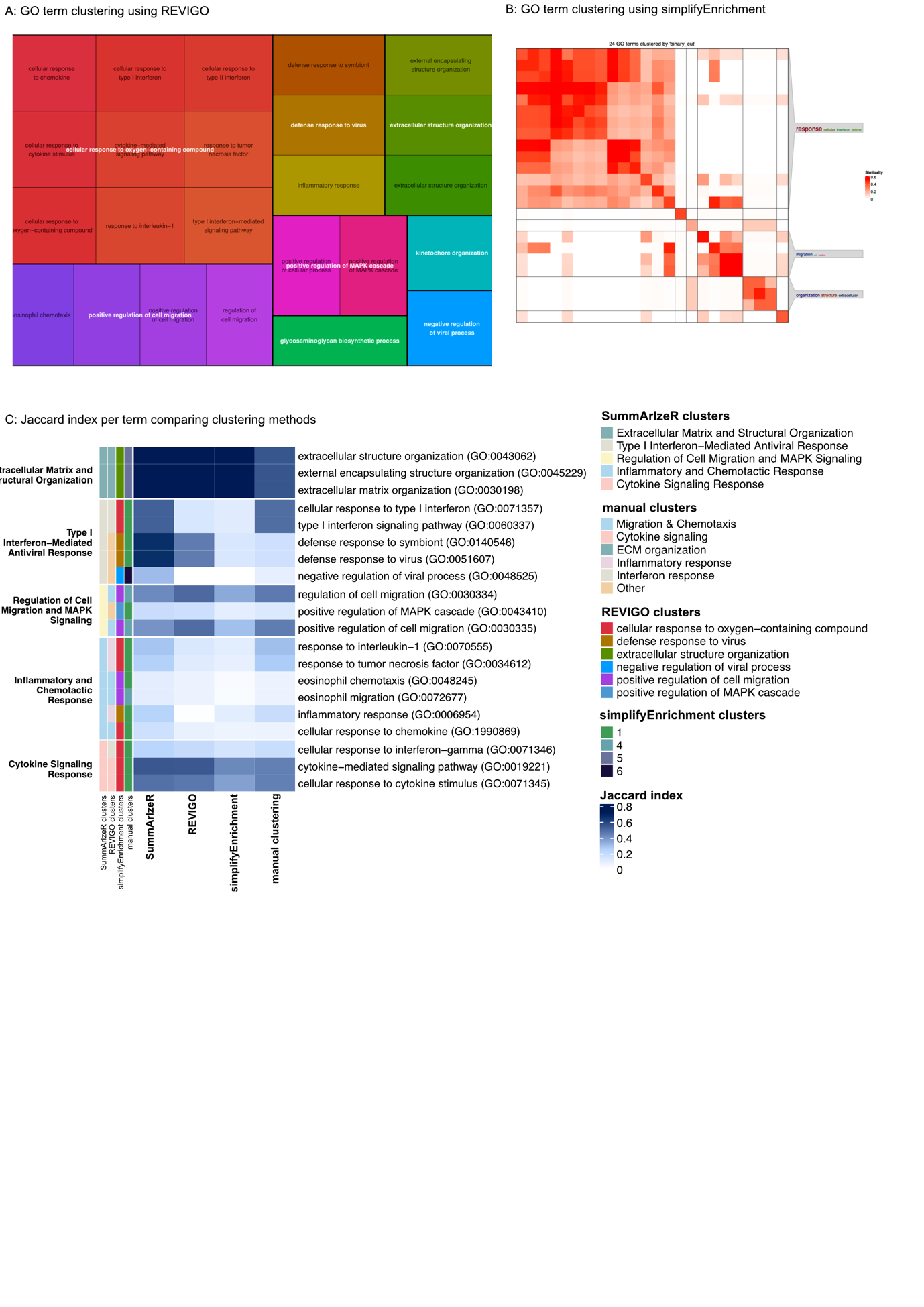


**Supplementary figure 1: A.** Automated clustering of GO terms corresponding to the analysis described in Kugler et al. using REVIGO, including parent-term annotation. **B.**Automated clustering of GO terms corresponding to the analysis described in Kugler et al. using simplifyEnrichment, including word cloud annotation.**C.** Benchmarking of SummArIzeR clustering (Figure 3B) against manual clustering (Figure 3A), REVIGO, and simplifyEnrichment. Cluster coherence was quantified using the Jaccard index, calculated for each term as the overlap between its gene set and the gene sets of the remaining terms within the same cluster (clusters containing more than one term).


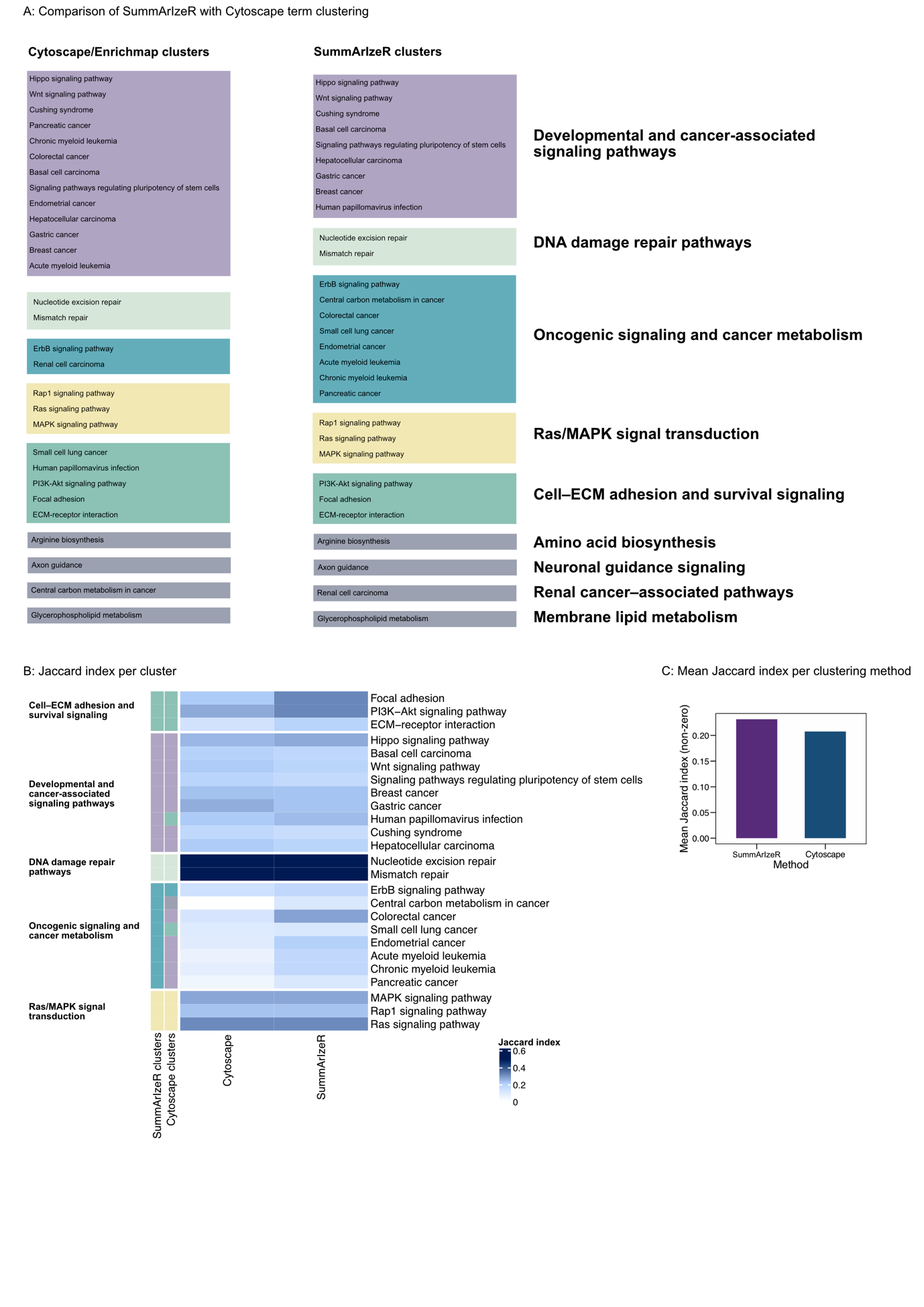


**Supplementary figure 2:** Validation of SummArIzeR term clustering using additional datasets. **A.** Automated clustering of KEGG terms with Cytoscape (EnrichMap), as described in Della Chiara et al., and SummArIzeR clustering of the same terms. **B.** Jaccard index per term comparing SummArIzeR and Cytoscape (EnrichMap). The Jaccard index was calculated for each term as the overlap between its gene set and the gene sets of the remaining terms within the same cluster (clusters containing more than one term).

**Supplementary table 2:** Cluster annotation of term clusters described in Fang et al. using parent terms, compared to SummArIzeR and word cloud annotations.

| **Term** | **Cytoscape annotation** | **SummArIzeR annotation** | **Word cloud annotation** |
| --- | --- | --- | --- |
| Antigen processing and presentation | Antigen processing and presentation | Antigen Presentation and Immune Response | antigen / presentation / processing |
| HIF-1 signaling pathway | HIF-1 signaling pathway | Cancer Metabolism and Hypoxia Signaling | cancer / carbon / carcinoma / cell / central |
| Renal cell carcinoma |  |  |  |
| Central carbon metabolism in cancer |  |  |  |
| Fructose and mannose metabolism | Fructose and mannose metabolism | Carbohydrate Metabolism | fructose / gluconeogenesis / glycolysis / mannose / metabolism |
| Glycolysis / Gluconeogenesis |  |  |  |
| Gap junction | Gap junction | Cell-Cell Communication (Gap Junctions) | gap / junction |
| AGE-RAGE signaling pathway in diabetic complications | AGE-RAGE signaling pathway in diabetic complications | Diabetic Complications and AGE-RAGE Signaling | gerage / complications / diabetic / pathway / signaling |
| nucleotide-excision repair (GO:0006289) | DNA replication | DNA Repair and Replication Processes | dna / nucleotideexcision / repair / replication |
| DNA replication |  |  |  |
| Mitophagy | Mitophagy | Mitochondrial Quality Control (Mitophagy) | mitophagy |
| Proteasome | Proteasome | Neurodegenerative and Protein Degradation Pathways | disease / ataxia / parkinson / prion / proteasome |
| Spinocerebellar ataxia |  |  |  |
| Prion disease |  |  |  |
| Parkinson disease |  |  |  |
| Pyrimidine metabolism | Pyrimidine metabolism | Nucleotide Metabolism (Pyrimidine Pathways) | metabolism / pyrimidine |
| Ferroptosis | Ferroptosis | Regulated Cell Death (Ferroptosis) | ferroptosis |
| p53 signaling pathway | p53 signaling pathway | Tumor Suppressor Signaling (p53 Pathway) | pathway / signaling |
